# Parvovirus B19-Associated Myocarditis: A Literature Review of Pediatric Cases

**DOI:** 10.7759/cureus.21726

**Published:** 2022-01-30

**Authors:** Stergiani Keramari, Alexandros Poutoglidis, Stefanos Chatzis, Michael Keramaris, Christos Savopoulos, Georgia Kaiafa

**Affiliations:** 1 Second Department of Paediatrics, University General Hospital of Thessaloniki AHEPA, Thessaloniki, GRC; 2 Department of Otorhinolaryngology-Head and Neck Surgery, George Papanikolaou General Hospital, Thessaloniki, GRC; 3 Department of Surgery, 251 Air Force General Hospital, Athens, GRC; 4 Department of Cardiology, General Hospital of Kastoria, Kastoria, GRC; 5 First Department of Propaedeutic and Internal Medicine, University General Hospital of Thessaloniki AHEPA, Thessaloniki, GRC

**Keywords:** treatment choices, infection rates, pediatrics, myocarditis, parvovirus b19

## Abstract

Parvovirus B19 (B19V) infection may lead to myocarditis, a life-threatening condition in pediatric patients. In this review, we aim to present published pediatric cases of B19V-associated myocarditis in order to understand the deep complex connections and draw useful conclusions. We performed a comprehensive search of MEDLINE, Science Direct, and Google Scholar electronic databases. A total of 32 cases were included in our study. The most common presenting symptom was tachycardia in 22/32 patients (68.7%), followed by tachypnoea (21/32, 65.6%), fever, and rash (12/32, 37.5% for both of them). Cardiac arrest, loss of consciousness, and systemic infection were associated with the worst prognosis, with statistically significant differences (p-value 0.001, 0.02, 0.001. respectively). A percentage as high as 90.4% of patients with left ventricular (LV) dysfunction and reduced ejection fraction (EF) were discharged. Twelve patients required ventilatory support, five required extracorporeal membrane oxygenation (ECMO), and three underwent heart surgery. Treatment with immunosuppressive agents and immunoglobulin was found to be beneficial for patients (p-value 0.006 and 0.004, respectively). In conclusion, B19V myocarditis has high mortality rates in children. There is no specific antiviral treatment for B19V infection and therapeutic strategies for myocarditis aim to delay the worsening of heart failure and to preserve the LV function. Inotropic drugs, diuresis, ventilatory support, Intravenous immunoglobulin (IVIG), and immunosuppressive therapy seem to help the recovery of the myocardium in children with LV dilation, dysfunction, and reduced EF. Children with cardiac arrest, arrhythmias, and loss of consciousness have the worst prognosis.

## Introduction and background

Viral myocarditis is an inflammatory process of the myocardial tissue with high morbidity and mortality rates in children. Viruses that are responsible for this inflammation are parvovirus B19 (B19V), human herpesvirus-6 (HHV-6), Epstein-Barr virus (EBV), coxsackievirus B, cytomegalovirus (CMV), and influenza virus. One of the most common causes of viral myocarditis is B19V. Parvoviruses are the smallest of all viruses (18 to 26 nm in diameter), non-enveloped single-stranded DNA viruses, of the family Parvoviridae, the sub family Parvovirinae. B19V is the only member, of the Parvoviridae family, that is known to be pathogenic in humans. Parvoviruses are widespread viruses. Seropositivity in children is 5%-10% among the ages 2-5 years, 50% in 15-year-old children and touching 90% in adults older than 60 years [1-3]. B19V is known for erythema infectiosum or fifth disease with a wide range of manifestations. In adults, the rash is less characteristic. Infection in healthy adults can cause acute symmetric polyarthropathy [4-7]. In patients with either short red blood cells life span or hereditary hematologic conditions like spherocytosis, sickle cell disease, thalassemia, or lack of G6PD enzyme, B19V infection may be manifested as transient aplastic crisis [8-10]. In healthy individuals, the long life span of erythrocytes causes the temporary suppression of erythropoiesis lasting about two weeks, due to neutralizing antivirus antibodies [11]. B19V infection could appear during pregnancy, with a wide range of clinical manifestations, such as hydrops fetalis or the development of congenital anemia [12-13]. Infection occurs via P-antigen cellular receptor and mechanisms leading to myocarditis are related to the primary infection or to autoimmune-mediated inflammation [14-15]. Myocarditis may lead to full recovery or reduction of left ventricular (LV) function and dilated cardiomyopathy, a disease frequently requiring surgery. Therapeutic strategies are controversial and antiviral treatment for B19V has not yet been approved. The aim of therapeutic management is to preserve LV function [16-18].

## Review

We conducted a comprehensive search in MEDLINE, Science Direct, and Google Scholar electronic databases, covering the time span between October 12, 2021 to December 3, 2021 - ("Parvovirus B19" OR "parvovirus infection") AND ("myocarditis" OR '' heart inflammation'') AND (children OR pediatric population OR age 0-18 years old). We performed an electronic search of current literature of pediatric case reports with diagnosed viral myocarditis with B19V that have been reported since 1997. Studies must include the following criteria: (1) patients’ age ranges 6 months-18 years old, (2) presenting symptoms and exam findings, (3) initial laboratory values (troponin, C-reactive protein, white blood cells, and hemoglobin), (3) electrocardiogram findings, (4) echocardiographic findings and (5) therapeutic approach. All cases were screened by two authors according to the previous descriptions. A number of 32 pediatric cases were identified. Taking into account that this is a review of published case reports, neither ethics approval nor informed consent was needed. Exclusion criteria were the following: (1) adult patients (age above 18 years old) (2) immunocompromised patients, (3) duplicates, (4) case series, and (5) conference abstracts. Both inclusion and exclusion criteria were determined before the commencement of the literature search.

Fisher’s exact test was used for categorical variables. We compared clinical characteristics, cardiac findings and treatment between survivals and deaths. Student’s t-test was used for continuous variables like the mean age of patients. Continuous data approximating the normal are presented with standard deviation. The graphs were created using GraphPad Prism software version 9.3.0 (GraphPad Software, La Jolla, CA). A p-value of < 0.05 was considered to be statistically significant.

Results

**Figure 1:**
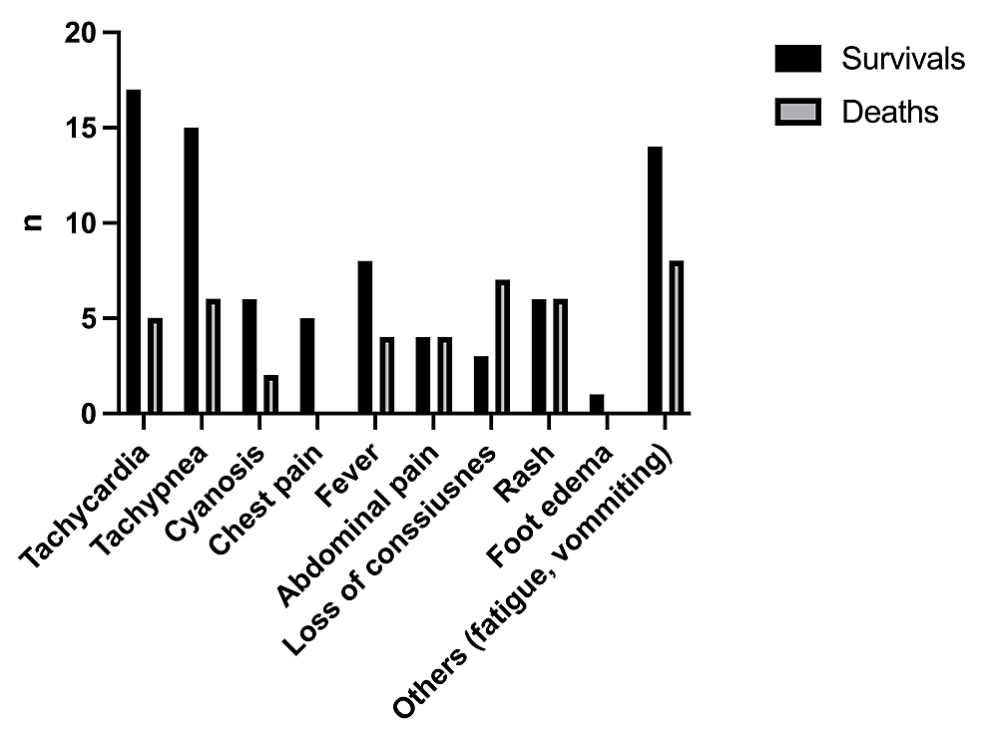
Outcomes in relation to presenting symptoms

In our review, 32 published cases with B19V myocarditis, between the years 1997 and 2020, were included. Patients’ median age was of 5.8 years +/- 5.2 with a range: (7 months-18 years). The most common presenting symptom was tachycardia in 22/32 of the patients (68.7%), followed by tachypnoea (21/32, 65.6%), fever, and rash (12/32, 37.5% for both of them). Ten patients presented unconsciousness 31.5% (three of them improved and seven died). Some of the patients presented with atypical symptoms like fatigue, vomiting, and joint edema (Figure 1). Laboratory biomarkers were consistent with myocarditis in 25/32 patients. A troponin median value was 11.4 μg/L and serum glutamic-oxaloacetic transaminase (SGOT) was 917 U/L. C-reaction protein and white blood cells (WBC) levels were increased in 13/32 patients tested. Anemia was observed in 14/32 children, with a mean value of 9.5 g/dL. In seven out of all patients, no laboratory tests were mentioned (Table 1).

**Table 1:** Characteristics and diagnosis of patients with parvovirus B19 myocarditis SGOT: Serum glutamic-oxaloacetic transaminase, CRP: C-reaction protein, WBC: white blood cells, Hb: Hemoglobin, PCR: polymerase chain reaction.

Patients’ characteristics	(n = 32 patients)
Median age: months (range)	5.8 years (7m-18y)
Male (%)	15 (46.8%)
Female (%)	17 (53.1%)
Other conditions	
Co-infection	4 (12.5%)
Herpes Simplex virus	2 (6.25%)
Sars-Cov-2	1 (3.12%)
Bacterial pneumonia	1 (3.12%)
Underlying condition-Hereditary disease	
Merosin-deficient congenital muscular dystrophy	2 (6.25%)
Sickle cell disease	1 (3.12%)
Systemic infection	6 (18.7%)
Laboratory (mean, range)	
CRP (mg/dl)	21.7 (0.09- 17.2)
WBC (cells/ml) elevanted	16313 (5000 - 32920)
Hb (g/dl) reduced	9.5 (2.7-14)
SGOT elevated (U/L)	917 (31-5810)
Troponin elevated (μg/L)	11.4 (0.13-79)
Diagnosis Parvovirus B19 -associated myocarditis	
Biopsy	20 (62.5%)
Blood test (PCR/ Serological test)	12 (37.5%)

Electrocardiogram (ECG) findings were mentioned in 20 pediatric patients. Tachycardia was recorded in all of them. The ECG findings were the following: ST-segment elevation (10/32, 31.5%), T wave inversion (4/32, 12.5%), low voltage QRS complexes (2/32, 6.25%). Arrhythmias were noticed in 5/32 of patients (15.6%) and two of them died. All patients with cardiac arrest did not recover (p-value 0.001). In a percentage of 65.6%, (21/32) echocardiographic findings showed LV dysfunction with reduced ejection fraction (EF). Four patients presented with impairment in both left and right heart chambers. In two patients, a thrombus in the LV apex was visualized, and after that, they were discharged (Table 2). Diagnosis of B19V myocarditis was established in patients with polymerase chain reaction (PCR) in a myocardial tissue biopsy (20/32, 62.5%) or with molecular assays in blood products (immunoassays and PCR). Most of biopsies were post-mortem (12/32, 37.5%). 

**Table 2:** Clinical manifestations, cardiac findings and therapeutic support of parvovirus B19 infection in patients with myocarditis LV: left ventricle, EF: ejection fraction, MV: mitral valve, ECMO: extracorporeal membrane oxygenation

Patients' Outcome	Deaths (n=12)		Alive (n=20)		Total (n=32)		p value
Age	5.1 y mean age +/- 2.9		6.2y mean age +/- 5.9		5.8 y mean age +/- 5.2		0.55
Male	4	33.3%	11	55.0%	15	46.8%	0.29
Symptoms							
Chest pain	0	0.0%	5	25.0%	5	15.6%	0.13
Fever	4	33.3%	8	40.0%	12	37.5%	1.00
Abdominal pain	4	33.3%	4	20.0%	8	25.0%	0.43
Loss of conssiusnes	7	58.3%	3	15.0%	10	31.5%	0.018
Rash	6	50.0%	6	30.0%	12	37.5%	0.28
Foot edema	0	0.0%	1	5.00%	1	3.12%	1.00
Others (fatigue, joint edema,vommiting among others)	8	66.6%	14	70.0%	22	68.7%	1.00
Clinical examination							
Tachycardia	5	41.6%	17	85.0%	22	68.7%	0.24
Tachypnea	6	50.0%	15	75.0%	21	65.6%	0.25
Cyanosis	2	16.6%	6	30.0%	8	25.0%	0.67
Electrocardiogram							
ST-elevation / changes	1	8.33%	9	45.0%	10	31.2%	0.049
T-wave inversion	0	0.0%	4	20.0%	4	12.5%	0.27
Low voltage QRS complexes	0	0.0%	2	10.0%	2	6.25%	0.52
Cardiac arrest	8	66.6%	0	0.0%	8	25.0%	0.001
Arrythmias	2	16.6%	3	15.0%	5	15.6%	1.00
Echocardiogram							
LV dilation	3	25.0%	13	65.0%	16	50.0%	0.065
LV dysfunction	2	16.6%	19	95.0%	21	65.6%	<0.001
EF reduced	2	16.6%	19	95.0%	21	65.6%	<0.001
Right Chamber dilated/ dysfanction	2	16.6%	2	10.0%	4	12.5%	0.061
Pericardial effusion	0	0.0%	3	15.0%	3	9.3%	0.27
Cardiac tamponade	0	0.0%	1	5.00%	1	3.12%	1.00
Others (MV regurgitation, thrombus in LV apex)	0	0.0%	4	20.0%	4	12.5%	0.27
Therapy							
Ventilatory support	3	25.0%	9	45.0%	12	37.5%	0.45
ECMO	2	16.6%	3	15.0%	5	15.6%	1.00
Surgery (Pericardial drainage / Heart transplantion )	1	8.33%	2	10.0%	3	9.3%	1.00
Others (resucitation)	8	66.6%	1	5.00%	9	28.1%	<0.001
Systemic infection	6	50.0%%	0	0.0%	6	18.7%	0.001

**Figure 2:**
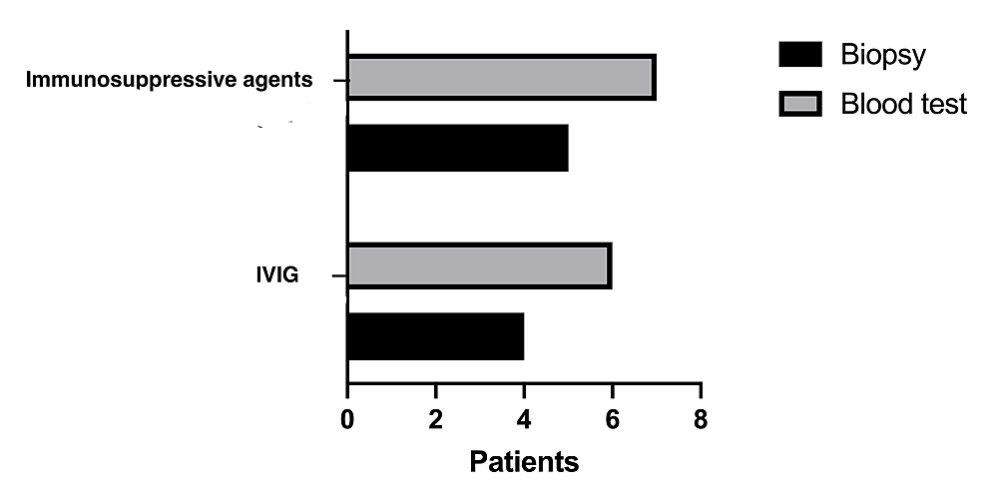
Diagnosis of parvoviral B19 myocarditis and treatment with immunosuppressive agents and intravenous immunoglobulin (IVIG)

The outcome was survival for 20/32 (62.5%) of patients and death occurred in a percentage as high as 37.5% (12/32). Therapeutic strategies in patients with impaired cardiac function included ventilatory support, diuresis, inotropic agents, beta-blockers, and immunoglobulin or immunosuppressive factors. Patients with poor recovery dealt with surgery (heart transplant or implant assist device). Twelve patients were treated with ventilation support (12/32, 37.5%). Five patients were supported with extracorporeal membrane oxygenation (ECMO) and two of them died. Resuscitation efforts were done in nine patients, but eight of them did not recover. Treatment with medications, included diuretics (10/32, 31.2%), inotropic agents (17/32, 53.1%), beta-blockers, or angiotensin-converting enzyme (ACE) (both 3/32, 9.3% for both of them). Immunosuppressive agents, like prednisone, azathioprine, cyclosporin, were administered in 12/32 (37.5%) patients and immunoglobulin in ten of them. Immunosuppressive agents are correlated with good prognosis, although 6/12 patients received this therapy without myocardial biopsy performed (Figure 2). In addition, systemic infection from B19V occurred in 6/32 children and co-infections have diagnosed in four patients. Two siblings with Merosin-deficient congenital muscular dystrophy and impaired LV function developed B19V associated myocarditis, one of them did not recover. 

Discussion

Myocarditis is a life-threatening condition. Myocarditis may present with nonspecific symptoms such as rash, abdominal pain, vomiting, fever, fatigue, or with cardiac symptoms such as chest pain, palpitations, dyspnea. One of the most common causes of viral myocarditis is B19V. Transmission of B19V occurs via the respiratory route, through blood products, and vertically via placenta from mother to fetus [1-3]. In healthy children, the main clinical symptom of B19V is a rash characterized as “slapped cheek” rash, known as fifth disease, and is combined with other nonspecific flu-like symptoms. The vast majority of cases, in immunocompetent hosts, are self-limited. It is known that B19V has tropism for erythroid progenitor cells. B19V is cytotoxic to host cells leading to hemolysis and the suppression of erythropoiesis. Their entry to erythroid progenitor cells occurs via P-antigen cellular receptor, which is present in the liver, placenta, endothelial cells of the coronary artery, and myocardial cells. The mechanisms that contribute to cardiac failure, due to myocarditis and liver dysfunction, are not clear and may be related to the primary infection P receptor-mediated virus endocytosis or to autoimmune-mediated inflammation [12-15]. Myocarditis is diagnosed and classified according to Dallas criteria on histopathological findings in endomyocardial biopsy [16]. Most patients’ laboratory findings are abnormal. These include elevated troponin, C-reactive protein, B-type natriuretic peptide, white blood cells, and creatinine kinase. Negative results do not rule out the diagnosis. Ventricular tachycardia is a rare life-threatening manifestation of myocarditis in less than 5% of patients [16-19]. Most frequently, ECG findings in patients are sinus tachycardia, ST-segment changes, prolonged QT, T wave inversions, or arrhythmias. Echocardiography may help to the diagnosis and show nonspecific findings such as left and/or right ventricular dysfunction, ventricular dilation, thickened ventricular wall, ventricular thrombus, or pericardial effusion. Most of the patients who developed myocarditis have mild LV dysfunction with mild reduced RF of 40%-50% and may have a full recovery. Patients with severe symptoms of heart failure and EF less than 35% may have a bad prognosis, in some cases [18].

Myocardium magnetic resonance is a non-invasive method to characterize the inflamed myocardium, identifying edema, early and late gadolinium enhancement. Diagnosis of parvoviral infection is based on detection of specific antibodies against B19V by enzyme-linked immunofluorescence assays, or on detection of viral DNA with the use of PCR [12]. High viral loads are associated with cardiac inflammation and may also be linked to short symptom duration. Myocardial viral loads > 316 000 copies/µg were detected in patients with acute B19V-associated myocarditis. Although, myocardium viral persistence is corelated with progressive cardiac dysfunction. The first step of the myocarditis therapeutic approach is management with oxygen and fluid status control. Patients with symptoms such as chest pain, normal cardiac enzymes, and normal LV function should be managed similar to pericarditis with colchicine or non-steroidal anti-inflammatory drugs (NSAIDs). There is no specific antiviral treatment for B19V infection. In persistent infection in immunocompromised individuals, administration of immunoglobulin reduces the viral load leading to resolution of anemia although supportive care with transfusion is often required in aplastic crisis [13-14]. In patients with heart failure symptoms, the aim is to preserve LV function and EF. Therapy includes beta-blockers, diuretics, ACE inhibitors, and/or angiotensin-II receptor blockers. However, beta-blockers are not recommended in patients with heart block or acutely decompensating phase of illness. In patients with heart failure symptoms and acutely decompensating phase of illness, inotropic support and/or mechanical circulatory support are fundamental. Digoxin may be useful for maintaining clinical stability in acute heart failure, due to positive inotropic and negative chronotropic mechanism of action in myocardial cells but should be used with caution and in low doses. Patients with poor recovery should be managed with surgery such as heart transplantation and implant assist device. The presence of arrhythmias is associated with a high mortality rate. Antiarrhythmic drugs should be used for the control of arrhythmias with caution due to negative inotropic action. Symptomatic bradycardia or heart block should be managed with a pacemaker and ventricular tachycardia with amiodarone [19-20]. Immunosuppressive therapy is controversial in the treatment of viral myocarditis. Immunosuppressive agents used are prednisone, azathioprine, cyclosporine. Mason et al. showed that patients experienced no benefit of immunosuppressive threapy [19]. Cooper et al. showed that they are beneficial in patients with giant cell and granulomatous myocarditis. Immunosuppressive agents were found to be beneficial for patients with dilated cardiomyopathy months after viral infection, with improved LVEF [20-26]. IVIG is recommended in patients with myocarditis due to antiviral and immunomodulating effects. Patients who recover from viral myocarditis should follow-up with echocardiographic studies to evaluate the ventricular function and LV dimension [24,27-28].

This is a literature review of 32 reported pediatric cases of B19V-associated myocarditis [29-53]. We found that immunosuppressive drugs and immunoglobulin administration may lead to good outcomes in pediatric patients (p-value 0.006 and 0.004, respectively) but this could be related to a limited number of cases. The LV dysfunction was not a poor prognostic factor in those infections. We found statistically significant differences for the outcome with respect to systemic infection, loss of consciousness, and cardiac arrest. In most of the survival cases, LV function returned to normal, and in one case, the echocardiography showed an improvement in LV function with persistence of the thrombus in the LV apex.

There are limitations to our study. The evidence obtained comes only from a limited number of case reports in the literature. Future studies are needed to validate the results of the present study. Small patient groups may not have been powered to determine the statistical significance. Furthermore, in patients with cardiac arrest and not effective resuscitation efforts, the previous LV function was unknown, and most episodes happened unexpectedly when the children were in good clinical condition. In some articles, laboratory biomarkers, ECG, echocardiography findings, treatment strategies, and time to LV normalization were not available. Despite our efforts, we were not able to retrieve this information. Another limitation is that case reports in literature may present patients with unusual characteristics and worse outcomes. All these could alter the results.

## Conclusions

B19V myocarditis has high mortality rates in children. Parvoviruses are widespread, with half of the adolescents being seropositive. In our review, the 32 patients with myocarditis were B19V positive, tested by serological tests and PCR from whole blood or endomyocardial tissue biopsies. Therapeutic strategies, like inotropic drugs, diuresis, ventilatory support, delayed the worsening of heart failure and preserved the LV function. In recent literature, the use of immunosuppressive therapy is controversial; however, in our study, it seems beneficial leading to the recovery of the myocardium with a statistically significant difference. Based on our findings, children with cardiac arrest, loss of consciousness, and systemic infection have the worst prognosis. There is a need for future prospective randomized studies and for further investigation in immunosuppressive treatment, therapeutic strategies, and a better understanding of the B19V B19 myocarditis.
